# Immunotherapy for Ovarian Cancer: Adjuvant, Combination, and Neoadjuvant

**DOI:** 10.3389/fimmu.2020.577869

**Published:** 2020-10-06

**Authors:** Chang Yang, Bai-Rong Xia, Zhao-Cong Zhang, Yong-Jian Zhang, Ge Lou, Wei-Lin Jin

**Affiliations:** ^1^ Department of Gynecology Oncology, Harbin Medical University Cancer Hospital, Harbin, China; ^2^ Department of Gynecology Oncology, The First Affiliated Hospital of USTC, Division of Life Sciences and Medicine, University of Science and Technology of China, Hefei, China; ^3^ Department of Instrument Science and Engineering, Institute of Nano Biomedicine and Engineering, Shanghai Engineering Center for Intelligent Diagnosis and Treatment Instrument, Key Laboratory for Thin Film and Microfabrication Technology of Ministry of Education, School of Electronic Information and Electronic Engineering, Shanghai Jiao Tong University, Shanghai, China; ^4^ National Center for Translational Medicine, Collaborative Innovational Center for System Biology, Shanghai Jiao Tong University, Shanghai, China

**Keywords:** ovarian cancer, immunotherapy, tumor microenvironment, combination therapy, immune checkpoint inhibitors (ICI)

## Abstract

Ovarian cancer is the most lethal gynecologic malignancy. Surgery and chemotherapy are the primary treatments for ovarian cancer; however, patients often succumb to recurrence with chemotherapeutic resistance within several years after the initial treatment. In the past two decades, immunotherapy has rapidly developed, and has revolutionized the treatment of various types of cancer. Despite the fact that immunotherapy response rates among ovarian cancer patients remain modest, treatment with immune checkpoint inhibitors (ICIs), chimeric antigen receptor (CAR)- and TCR-engineered T cells is rapidly developing. Therapeutic efficiency could be improved significantly if immunotherapy is included as an adjuvant therapy, in combination with chemotherapy, radiation therapy, and the use of anti-angiogenesis drugs, and poly ADP ribose polymerase inhibitors (PARPi). Newly developed technologies that identify therapeutic targets, predict treatment efficacy, rapidly screen potential immunotherapy drugs, provide neoadjuvant immunotherapy, and utilize nanomedicine technology provide new opportunities for the treatment of ovarian cancer, and have the potential to prolong patient survival. However, important issues that may hinder the efficacy of such approaches, including hyperprogressive disease (HPD), immunotherapy-resistance, and toxicity of the treatments, including neurotoxicity, must be taken into account and addressed for these therapies to be effective.

## Background

According to the World Health Organization, it is estimated that the global incidence of ovarian cancer will be 308,069 patients and the total mortality from ovarian cancer will be 193,811 patients, in 2020 (1). The standard treatment for ovarian cancer includes surgery followed by platinum-based chemotherapy. Currently, the five-year survival rate of ovarian cancer is approximately 47%, predominately due to relapse and chemoresistance (2). In recent years, poly-ADP-ribose polymerase (PARP) inhibitors (PARPi) have seemed to be a promising option to treat cases that have BRCA mutations and BRCA wild-type EOC tumors (3). Despite an increase in the number of clinical trials, and a growing number of approved drugs, the therapeutic effects of PARPi are limited, as treatment only extends survival by a few months, and does not provide any long-term benefit (4). Resistance to PARPi in BRCA1/2-mutated tumors suggests that this therapy may not be as effective as previously thought (5–7). The most frequently reported mechanism of resistance is (partial) restoration of homology directed DNA repair. Understanding the mechanisms underlying this resistance and combining therapies may be useful to counteract PARPi resistance. A combination of Bevacizumab and a platinum/taxane-based chemotherapy has been recommended as a first-line therapy for ovarian cancer. Results suggest that progression free survival is prolonged by 3.5-months, whereas no significant differences in overall survival were observed. Thus, a more effective treatment regime is needed to prolong the overall survival of patients.

**Figure 1:**
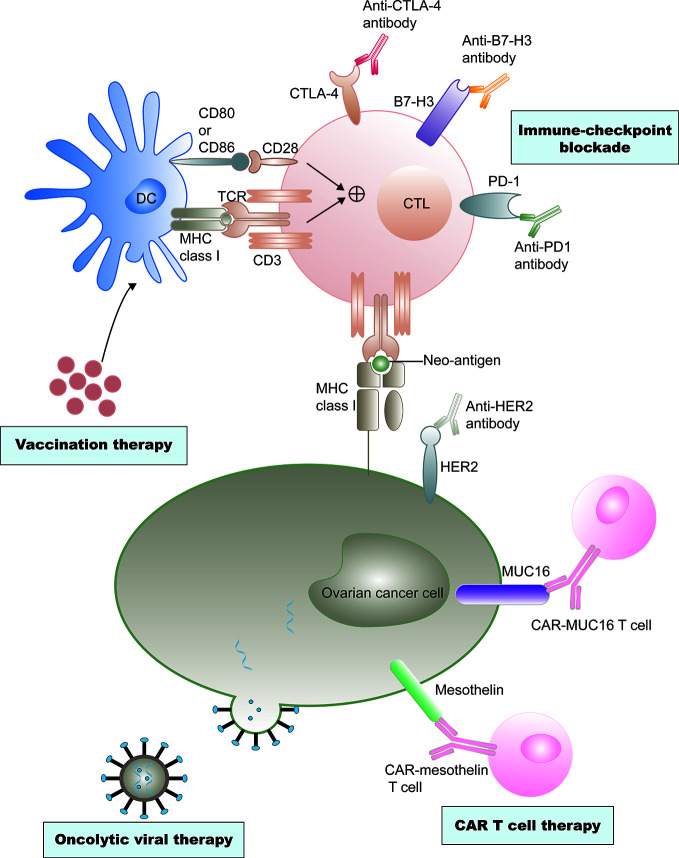
Current immunotherapy treatments of ovarian cancer. Ovarian cancer vaccine therapy is based on dendritic cell (DC)-mediated presentation of neo-antigens derived from malignant ovarian cancer cells to T cells through MHC class I–T cell receptors (TCRs) and a co-stimulation signal of CD80 and/or CD86-CD28 interactions. CTLs are subsequently activated to destroy tumor cells. However, tumor cells often escape immune destruction by CTLs through upregulation of immune checkpoint ligands, such as programmed cell death 1 ligand 1 (PD- L1), that can bind the programmed cell death protein 1 (PD-1) on the CTLs. Immune checkpoint inhibitors could effectively prevent this effect. Antibody-mediated blockade of cytotoxic T lymphocyte protein 4 (CTL A-4), an inhibitory immune- checkpoint molecule that binds CD80 and CD86 and prevents their interaction with CD28, can promote T cell priming by DCs. Similarly, anti–B7-H3 antibody-mediated blockade could neutralize T cell exhaustion in patients with ovarian cancer. Neo-antigens, including human epidermal growth factor receptor 2 (Her2), cancer antigen 125 (MUC16), and mesothelin, are also presented on tumor cell surfaces independent of MHC class I receptors, and these neo-antigens are specific targets of chimeric antigen receptor (CAR) T cell therapies. Genetic engineering is also applied to produce viruses that selectively infect or replicate in tumor cells, and finally destruct tumor cells. Cell destruction can also promote immunogenic tumor cell death, which can active antigen presentation and an adaptive antitumor immune response.

In the past two decades, immunotherapy has developed rapidly, revolutionizing the treatment of various types of cancer. Recently, immune checkpoint inhibitors, including CTLA-4 and programmed cell death protein 1 (PD-1)/programmed cell death 1 ligand 1 (PD-L1) inhibitors, which reverse the signals from the immunosuppressive tumor microenvironment (TME), are being investigated as potential treatment modalities (8, 9). The application of oncolytic viruses, cancer vaccines, and adoptive cell therapies is advancing rapidly (10–12). Immunotherapies have also attracted significant attention in ovarian cancer therapy (**Figure 1**).

Epithelial ovarian cancers (EOCs) have been considered “immunogenic tumors”, non-spontaneous antitumor immune responses could be detected in the tumors, peripheral blood, and ascites of patients with EOCs (13). Immune cells in the tumors and ascites, including T and B lymphocytes, regulatory T cells (Tregs), natural killer (NK) cells, tumor-associated macrophages (TAMs), and myeloid-derived suppressor cells (MDSCs) play key roles in ovarian cancer. Fibroblasts and adipocytes in the tumor microenvironment may also affect the efficacy of immunotherapeutic and chemotherapeutic drugs (14).

In this review, we summarize recent advances in immunotherapy for ovarian cancer, including cancer vaccines, adoptive cell therapy (ACT), immune checkpoint inhibitors (ICIs), oncolytic viruses, and the immunosuppressive tumor microenvironment. We also introduce diverse treatment modalities that can be used in combination with immunotherapy. In addition, we briefly discuss the future directions of these therapies.

## The Immunosuppressive Tumor Microenvironment in Ovarian Cancer

**Figure 2:**
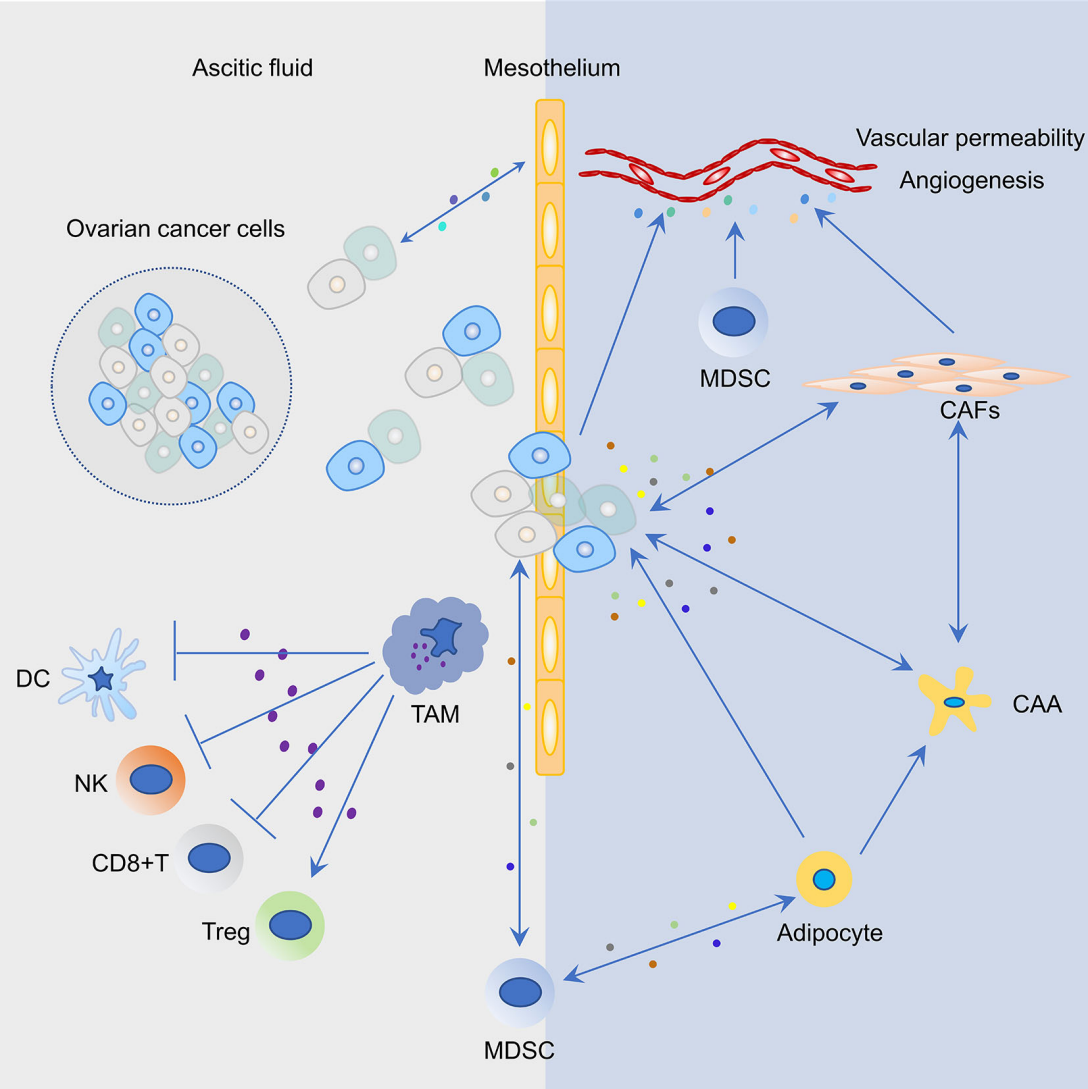
Representative cell types and their interactions in the tumor microenvironment (TME) of ovarian cancer. Cancer cells and mesothelium cells secrete pro-inflammatory cytokines and chemokines, including macrophage chemoattractant protein-1 (MCP1/CCL2), which recruits tumor associated macrophages (TAM) to the peritoneum. Increased infiltration of TAMs in the peritoneal TME not only promotes ovarian cancer cell invasion but also induces an immunosuppressive environment that suppresses the function of T cells, dendritic cells (DCs), and natural killer (NK) cells and activates the function of regulatory T cells (Tregs). Mesothelial cells are the first barrier to this process; however, the bidirectional cross-talk between cancer and mesothelial cells results in mesothelial clearance and invasion of the sub-mesothelial layers. Myeloid-derived suppressor cells (MDSCs) are attracted to the tumor site in response to growth factors and inflammatory cytokines/chemokines that are secreted by ovarian cancer cells. MDSCs could suppress T cell function, maintain the ovarian cancer stem cell pool, and interact with adipocytes. In the ovarian TME, blood vessel structure is modulated by a plethora of factors that are secreted by ovarian cancer cells, MDSCs, and cancer associated fibroblasts (CAFs). Molecular cross-talk between cancer cells and CAFs in the ovarian TME produce a pro-inflammatory TME and promote tumor progression. The bidirectional interaction between omental adipocytes and cancer cells promote dedifferentiation and reprogramming of adipocytes into cancer-associated adipocytes (CAAs). In turn, cancer cells absorbing fat rapidly promote tumor proliferation.

The immunosuppressive immune cells include myeloid-derived suppressor cells (MDSCs), Tregs, TAMs, cancer-associated fibroblasts (CAFs), and adipocytes (**Figure 2**). In order to develop effective immunotherapies against ovarian cancer, immunosuppressive networks within primary ovarian tumors, ascites, metastatic tumors, and related mechanisms must be considered.

### Suppressive Immunology of Ovarian Cancer

#### Myeloid-Derived Suppressor Cells

MDSCs consist of multiple immature myeloid cells that are elevated in pathological situations, and which weaken the efficacy of the T cell response (15, 16). In 2017, Horikawa et al. demonstrated that the number of MDSCs is increased by VEGF expression in ovarian cancer, which suppresses local immunity (17). Taki et al. have identified that Snail, a major transcription factor, could increase the expression of CXCR2 ligands and recruit MDSCs, thus resulting in poor prognoses (18). In 2019, a study using mouse EOC cells identified MDSCs as a driving factor for immunosuppression in a ID8-fLuc ovarian cancer mouse model (19).

#### Regulatory T Cells

Tregs directly or indirectly inhibit antitumoral responses, suggesting that Tregs are a primary means of tumor immune escape (20). As early as 2005, Sato et al. concluded that the number of intraepithelial CD8+ TILs and a high ratio of CD8+/Treg are associated with a positive prognosis in EOC (21). TGFβ, IL-10, and tumor-derived exosomes, which contain IL-10, TGFβ, IDO1, and PDL1/L2, are secreted from tumor cells and potentiate the differentiation of inhibitory Tregs, which is characterized by increased expression of FoxP3 and CTLA4 (22–24).

#### Tumor-Associated Macrophages

TAMs that infiltrate tumors and differentiate into macrophages are derived from the bone marrow. TAMs consist of both M1 and M2 type macrophages (25, 26). In EOCs, M2 type macrophages in the abdominal cavity and ascites are cancer promoting. Zhang et al. demonstrated that patients with upregulated overall or intra-islet M1/M2 TAMs ratios have an advanced five-year survival rate (27). Yin et al. identified that TAMs play a prominent role in promoting early peritoneal cavity metastasis of ovarian cancer *via* EGF secretion (28).

However, the regulation between cancer cells and TAMs is bidirectional. Ovarian cancer cells play a critical role in promoting M2 polarization of TAMs, and TAMs can result in cisplatin resistance *via* the enhancement of cellular stemness in cisplatin-sensitive cells (29).

#### Cancer-Associated Fibroblasts

Cancer-associated fibroblasts (CAFs) are the primary type of stromal cell, and express α-smooth muscle actin and fibroblast activation protein in ovarian cancer (30). CAFs are enhanced in ovarian cancer tumor cells, and therefore secrete high levels of hepatocyte growth factor, which facilitates tumor cell proliferation, chemoresistance, invasion, and migration though activation of the cMet/PI3K/Akt pathways and glucose-regulated protein 78 (31). CAFs produce pro-inflammatory cytokines, such as COX-2, CXCL1, CCL5, CXCL11, and IL-6, which increase tumor cell proliferation and EMT (32–36).

#### Adipocytes

Many studies have reported that obesity is associated with increased incidence and poor prognosis of ovarian cancer. Adipocytes in the omentum secret cytokines and chemokines, such as IL-6, IL-8, MCP-1, tissue inhibitor of metalloproteinases-1, and adiponectin, to promote transcoelomic metastasis and tumor progression (37, 38). Lipid metabolism has also been shown to shape the tumor microenvironment (TME), which could affect the treatment efficacy of immunotherapies. Strategies based on this knowledge have the potential to increase immunotherapeutic response and patient survival (39).

Since the production of immunosuppressive cytokines and chemokines is likely to be shaped by the intrinsic biologic properties of the tumor, therapeutic combinations that can minimize toxicity and maximize benefits, to eliminate EOC, are needed.

### Immunosuppressive Networks in Ovarian Cancer

Due to the immunosuppressive microenvironment of ovarian cancer, tumor-specific T cells generated by immunotherapy could not destroy tumors in EOC patients. Communication between tumor cells and other cells in the TME occurs *via* contact-dependent and -independent mechanisms. Tumor cells are in direct contact with cells in the extracellular matrix for contact-dependent mechanisms, whereas communication is achieved *via* soluble molecules such as cytokines, lipid mediators, and growth factors in contact-independent mechanisms (40). Ovarian tumors have been reported to recruit Tregs and myeloid-derived suppressor cells, which can inhibit the activation and efficacy of CD8+ effector cells (41, 42). Moreover, the stromal cells in the TME, such as MDSCs, Tregs, TAMs, CAFs, and adipocytes could be “educated” to facilitate and sustain cancer cells (43). Soluble factors in the TME function as a limiting factor for the maturation of local antigen-presenting cells, rendering them unable to generate costimulatory signals to effector cells, and consequently inducing the failure of T cell efficacy.

To conclude, the mechanisms of immunosuppressive networks of ovarian cancer include inhibition of CD8+ effector cells by Tregs, suppression of receptor PD-1 engaging by the ligand PD-L1; myeloid-derived suppressor cells and inhibitory cytokines (44).The immunosuppressive network is a significant obstacle in immunotherapy, which must be overcome to ensure the implementation of effective immunotherapeutic strategies.

## Current State Of Ovarian Cancer Immunotherapy

Most types of ovarian cancer immunotherapy treatment modalities are currently being tested in clinical trials (**Table 1**). Adaptive immunity in ovarian cancer is rapidly expanding to enhance dendritic cell (DC)-mediated presentation of ovarian cancer, predominantly by vaccination (45, 46) (**Figure 1**). Cytotoxic T lymphocytes (CTLs) are activated after recognizing tumor-associated antigens (TAAs), and particularly the neoantigens (47). Ovarian cancer tumor cells often evade destruction by CTLs through inhibitory signals in the tumor (48). Mucin 16, α-folate receptor, and mesothelin are being investigated as specific targets of genetic modification of chimeric antigen receptor T (CAR-T) cell therapies for ovarian cancer (49). Oncolytic viral therapy that utilizes genetic engineering is also being used to create viruses that selectively infect tumor cells and lead to tumor cell lysis (11). In this section, we have discussed the evolution of immunotherapies for ovarian cancer.

**Table 1:** Clinical trials of immunotherapy for ovarian cancer.

Immunotherapy treatment strategy	Not yet recruiting	Recruiting	Active, not recruiting
Autologous dendritic cell vaccination	NCT03905902		
Autologous dendritic cell vaccination + chemotherapy			NCT03657966
Autologous monocytes + peginterferon + interferon		NCT02948426	
Autologous T cells therapy			NCT02346747
			NCT01309230
Autologous T cells therapy + CD274 antibody			NCT02725489
Autologous T cells therapy + PD-L1 inhibitor			NCT03073525
cancer vaccine (V3-OVA)		NCT03556566	
CAR-T		NCT03638206	
CD274 antibody + chemotherapy			NCT02431559
CTLA-4 inhibitor + PARPi		NCT04034927	
		NCT02571725	
CTLA-4 inhibitor + CD274 antibody + chemotherapy		NCT03249142	
		NCT03277482	
HER-2/neu peptide vaccine			NCT00194714
Oncolytic vaccinia virus + chemotherapy + VEGF-A		NCT02759588	
PD1 inhibitor		NCT03755739	
	NCT03959761		
PD1 inhibitor + chemotherapy			NCT02608684
		NCT03914612	
		NCT03914612	
		NCT03989336	
	NCT03539328		
PD1 inhibitor + PARPi			NCT03824704
PD1 inhibitor + TLR-3 agonist +chemotherapy		NCT03734692	
PD1 inhibitor + CA125 antibody		NCT03100006	
PD1 + cancer vaccine	NCT03761914		
PD1+ cancer vaccine + chemotherapy	NCT03836352		
PD-L1 inhibitor + CD137 antibody		NCT02554812	
TCR		NCT03412877	
TIL		NCT01174121	
TLR-3 agonist + CA125 antibody			NCT03162562

PD-L1, programmed death-ligand 1; CAR-T, chimeric antigen receptor T cells; CTLA-4, cytotoxic T lymphocyte–associated protein 4; PARPi, poly ADP ribose polymerase inhibitors; PD1, programmed cell death protein 1; HER-2, human epidermal growth factor receptor 2; TLR-3, Toll-like receptor 3; VEGF-A, vascular endothelial growth factor A; TCR, T cell receptor; TIL, tumor-infiltrating lymphocytes.

### Immune Checkpoint Inhibitors

Successful immunotherapy for ovarian cancer relies on the stimulation of antigen-presenting cells, attenuating the immunosuppressive microenvironment, and bolstering effector T cell activity. The T cell–mediated immune response is regulated by inhibitory and stimulatory signals. Immune checkpoint receptors negatively regulate T cell activation and are critical to prevent over-activation. However, various types of tumors express immune checkpoints, leading to immune escape. Thus, immune-checkpoint blockade inhibitors play an important role in immunotherapy. To date, the most promising immune checkpoint inhibitors have been antibodies that block cytotoxic T lymphocyte antigen 4 (CTLA­4) and PD­1, which are expressed on T cells, or PD­ L1, which is expressed on certain immune cell subsets and is aberrantly expressed on tumor cells. The Food and Drug Administration–approved immune checkpoint inhibitors consist of a CTLA-4 antibody (Ipilimumab), PD-1 antibodies (Pembrolizumab and Nivolumab), and PD-L1 antibodies (Avelumab, Atezolizumab, and Durvalumab) (50). These ICIs have shown significant clinical benefits in multiple types of tumors, particularly melanoma (51). Little success has been found in the clinical use of checkpoint inhibitors in ovarian cancer, whose single-agent objective response rates in clinical trials are approximately 6–15% (52, 53).

CTLA-4 is a receptor on T cells that has the same set of ligands as costimulatory receptor CD28, but with a higher binding affinity, leading to competitive inhibition (54). Anti-CTLA-4 could block the binding of CTLA-4 and its ligands and prevent further inhibitory signal transduction, resulting in increased CD28-mediated co-stimulation.

The most commonly investigated immune checkpoint target in ovarian cancer is the PD-1/PD-L1 pathway. PD-1 is also expressed on T cells, and regulates the activation of effector T cells, mainly in the effector phase in peripheral tissue and the tumor microenvironment (55). After binding with its ligands, PD-L1 or PD-L2, PD-1 is phosphorylated and recruits the inhibitory phosphatase that can rapidly dephosphorylate CD28 and inhibit the co-stimulatory signaling pathway. Thus, an antibody targeting PD-1 could counteract its inhibitory effects. The efficacy of dual inhibition of PD-L1 and PD-L2 is more effective than anti–PD-1 or PD-L1 alone in various types of cancers that express both PD-L2 and PD-L1, including ovarian cancer (56). Exosomes expressing PD-L1 can repress anti-tumor immune responses (57). Developing a better understanding the mechanisms of exosomal PD-L1 in immune oncology is important, because inhibition of exosome production may be exploited as a potential new therapy.

Other potential targets of immune checkpoint blockage are listed in **Table 2**. B7-H3 is an immunosuppressive molecule that is expressed on tumor cells, but not host cells. The antitumor effects of B7-H3 and PD-1 blockade have been studied. Findings suggest that B7-H3, rather than PD-1 blockade, extends the median survival time of ID8 tumor-bearing mice with ovarian cancer. Collectively, B7-H3 may be utilized as a novel target in ovarian cancer patients that are not responsive to PD-L1/PD-1 inhibition (58). B and T lymphocyte attenuator is a novel inhibitory receptor, whose structure and function are similar to those of CTLA-4 and PD-1 (63). LAG-3 (CD223) is a cell surface molecule that is expressed on several immune cells, including activated T cells. LAG-3 has been shown to be involved in CD8+ T cell exhaustion (64). TIM-3 is a co-inhibitory receptor on IFN-γ-producing T cells, FoxP3+ Treg cells, and innate immune cells (macrophages and DCs). By binding with its ligands, TIM3 can suppress the immune response of these cells (65). The V-domain Ig suppressor of T cell activation, which is expressed on tumor cells, has been identified as a novel inhibitory immune-checkpoint protein in ovarian cancer (62). These novel immunosuppressive factors within the tumor microenvironment are promising targets for immunotherapy.

**Table 2:** Selective targets that block immune checkpoints in ovarian cancer.

Targets	Model	Result	Refs
B7-H3	ID8 OvCa mouse model	B7-H3 blockade prolonged the survival of ID8 tumor bearing mice.	(58)
BTLA	Ascitogenic mouse model	Inhibition of BTLA combined with chemotherapy can elevate immune activation and generate potent anti-tumor effects.	(59)
LAG-3	Mouse orthotopic tumor model	Dual blockade of PD-1 and LAG-3 can enhance T cell effector function, slow murine ovarian tumor growth, increase numbers of CD8+ T cells, and reduce the frequency of Tregs and MDSC in the peritoneal TME.	(60)
TIM-3	Fresh HGSC samples	TIM-3 constitutes a prognostically relevant biomarker of active and suppressed immune responses against HGSC.	(61)
VISTA	Intraperitoneal tumor mouse model	Anti-VISTA antibody prolonged the survival of tumor-bearing mice.	(62)

B7-H3, CD276; OvCa, ovarian cancer; BTLA, B- and T lymphocyte attenuator; LAG-3, lymphocyte-activation gene 3; MDSC, myeloid-derived suppressor cells; TME, tumor microenvironment; TIM-3, T cell immunoglobulin and mucin-domain containing-3; HGSC, high-grade serous carcinoma; VISTA, V- domain Ig suppressor of T cell activation.

Currently, in order to test the clinical response to ICI therapy, there is a need to identify patients who would be expected to benefit from this immunotherapy (66, 67). 5-methylcytosine and 5-hydroxymethylcytosine may act as prognostic and predictive biomarkers of ICI-sensitive cancers (68). Thus, exploration of multimodal predictive models of target assessment, tumor-intrinsic features, independent host features, and the tumor microenvironment are needed to optimize the treatment of ICIs in the future (66).

### Adoptive Cell Therapy

Adoptive cell therapy (ACT) is a promising strategy for the treatment of cancer, that utilizes the cells of the immune system to eliminate cancer. Currently, ACT can be classified into adoptive T cell therapy and other immune cell types, such as NK cells (69), cytokine-induced killer (CIK) cells (70), and macrophages (71). In this review, we primarily discuss adoptive T cell therapy. Adoptive T cell therapy that infuses the *ex vivo*–expanded tumor-specific T cells has shown promise as an immunotherapy treatment for cancer patients (72). Adoptive therapy with tumor-specific T cells consists of two major forms: genetic modification of T cells for expression of a specific T cell receptor (TCR) and CAR (73, 74). Both approaches have been tested in preclinical ovarian cancer models and have also been evaluated in early-phase clinical trials.

#### TCR-Engineered T Cells

Therapy with autologous T cells that have been genetically modified to express a cloned TCR directed toward a specific antigen has considerable potential for clinical application in cancer patients. Similar to endogenous T cells, these engineered cells are primed by recognition of the antigen in the context of the major histocompatibility complex (MHC), and can be negatively regulated by immunosuppressive signals in the TME.

TCR-engineered T cells have induced significant objective responses in the majority of treated patients. In ovarian cancer, two clinical trials to evaluate the efficacy of TILs (NCT02482090 and NCT01883297) are ongoing. Recently, Matsuda et al. have built a rapid and efficient process for the production of neoantigen-specific TCR-engineered T cells exploiting blood from an HLA-matched healthy donor. They successfully identified three neoantigen-specific TCRαβ pairs from 14 estimated neoantigen candidates, and also revealed the importance of careful validation for the specificity of TCRs against neoantigens (75).

#### Chimeric Antigen Receptor T Cells

CAR-T cell therapy is another strategy for antitumor treatment that provides recognition specificity to engineered T cells. The CAR molecule consists of an antigen-binding domain and a cytoplasmic signaling motif. The targets of CAR-T therapy in ovarian cancer are listed in **Table 3**. Phase I/II clinical trials are currently in progress in order to investigate CAR-T cells targeting MUC16 (NCT02498912), mesothelin (NCT01583686), and NY-ESO-1 (NCT01567891 and NCT02457650) for ovarian cancer. Recently, Garcia et al. demonstrated that T cells expressing the Müllerian inhibiting substance type 2 receptor (MISIIR)–specific CAR exhibited significant antigen specification reactivity and eliminated MISIIR overexpression in tumors *in vivo.* This group also conducted *in vitro* experiments and confirmed that without cytotoxicity to normal primary human cells, MISIIR CAR-T recognized various types of human ovarian and endometrial cancer cell lines (76).

**Table 3:** Clinical trials of CAR-T therapy in epithelial ovarian cancer.

Target antigen	Therapeutic compounds	Patients	NCT number
CD133	Anti-CD133-CAR vector-transduced T cells	Chemotherapy refractory or relapsed CD133-positive EOC	NCT02541370
EGFR	Anti-EGFR-CAR transduced autologous T cells	Chemotherapy-resistant or relapsed EOC with EGFR expression	NCT01869166
ErbB2/Her2	Anti-HER-2-CAR transduced autologous T cells	Chemotherapy-resistant or relapsed EOC with Her2 expression	NCT01935843
Folate receptor α	MOv-gamma CAR transduced PBL	Recurrent FR+ EOC	NCT00019136
Mesothelin	Anti-mesothelin CAR transduced T cell	EOC	NCT02159716
Mesothelin	Anti-mesothelin CAR transduced PBL	Metastatic or unresectable cancer that expresses mesothelin	NCT01583686
Mesothelin	Anti-mesothelin CAR transduced T cells	Refractory or relapsed mesothelin expressing tumor	NCT02580747
MUC16	4H11–28z/fIL-12/EGFRt + genetically modified T cells	EOC with MUC16 ecto antigen expression	NCT02498912

CAR, chimeric antigen receptor; EOC, epithelial ovarian cancer; EGFR, epidermal growth factor receptor; ErbB2, receptor tyrosine-protein kinase erbB-2; Her2, human epidermal growth factor receptor 2; MOv-gamma, monoclonal antibody MOv18; PBL, peripheral blood lymphocytes; MUC16, cancer antigen 125.

Despite their promising results in hematological malignancies, application of CAR-T cells in solid tumors has been limited by physical barriers and tumor heterogeneity (77, 78). Compared with liquid tumors, physical barriers between CAR-T cells and ovarian cancer cells inhibit solid tumor accessibility by CAR-T cells. To address this issue, local administration of CAR-T cells will improve T cell therapies (79).

With the development of synthetic biology techniques, more robust generation of TCR or CAR modified T cells is possible. Next generation TCR or CAR-modified T cells may increase the number of decoy receptors for inhibitory molecules (80, 81) or and it is possible that further clinical research and application of next generation approaches can be used to treat ovarian cancer.

### Cancer Vaccines

Cancer vaccines are used to strengthen tumor-associated antigen (TAA) presentation by APCs and spark TAA-specific CD8+ T cells to kill tumor cells. Vaccine-induced immune responses provide long-term immunologic memory. Cancer vaccines may be classified into cell-based vaccines, peptide/protein vaccines, epigenetic vaccines, and genetic vaccines (82). Among these, peptide/protein vaccines and cell-based vaccines are usually based on well-defined TAAs (51). TAAs in ovarian cancer can be classified into two categories: shared common TAAs and individually mutated neo-antigens. The shared antigens contain three types of antigens: overexpressed antigens, such as mesothelin, tissue-specific TAAs, and TAAs whose expression is generally restricted to male germline cells, such as NY-ESO-1 (83). The TP53 gene, which encodes the tumor suppressor p53, is commonly mutated in ovarian cancer. Malekzadeh et al. systematically studied intertumoral T cell responses to the eight most commonly mutated positions of P53. The findings suggest that TCRs were identified in mutated TP53 and may be candidates with which to evaluate targeted immune cancer therapies (84).

The immunogenicity of ovarian cancer is still ambiguous. Schumacher et al. demonstrated that ovarian tumors have a very heterogeneous and comparatively low mutational load, thus making immune recognition of neo-antigens uncertain (85). In the future, combinations of multiple immunotherapies will be necessary to effectively provoke immune responses and take advantage of the immunogenicity of ovarian cancer (83).

### Oncolytic Viruses

The use of oncolytic virus anticancer therapies has been considered as an independent treatment strategy that is separate from immunotherapy. Viruses can replicate in cancer cells, leading to subsequent destruction of the cells. However, oncolytic viral infections could generate antitumor immune responses. Viruses can stimulate the immune system through pathogen-associated molecular patterns and pattern recognition receptors, and viruses often activate macrophages through receptors (86).

Santos et al. established patient-derived *ex vivo* tumor cultures in order to investigate the possibility of using TNFα and IL-2 encoding oncolytic adenovirus to restore and enhance the tumor reactivity of TILs in the context of immunosuppressive human ovarian cancer (87). Oncolytic adenovirus Ad5/3-E2FD24-hTNFa-IRES-hIL2 was able to rewire the ovarian tumor microenvironment to heighten antitumor TIL reactivity.

In the future, it will be important to combine oncolytic viral therapies with other immunotherapy strategies to establish prolonged anticancer immune responses initiated by viral infection.

## Combination Therapies

In 2010, Weinberg summarized ten hallmarks of cancer, including proliferative signaling, evading growth suppressors, resisting cell death, enabling replicative immortality, inducing angiogenesis, activating invasion and metastasis, reprogramming energy metabolism, evading immune destruction, promoting genome instability and mutation, and activating tumor-promoting inflammation (88, 89). Almost all cancers have acquired similar capabilities during tumor progress *via* different mechanistic strategies. The hallmarks of cancers, especially deregulating cellular metabolism and evading immunological destruction, are an increasingly common problem faced by immunotherapies; therefore, the combination of immunotherapy with chemotherapy, radiation therapy, anti-angiogenesis drugs, and PARP inhibitors is essential.

### Chemotherapy in Combination With Immunotherapy

Chemotherapy is the cornerstone of the treatment of EOC, and can lead to the destruction of cancer cells and the release of immunogenic molecules (90). Platinum-based chemotherapy can interfere with the STAT6-mediated immunosuppression in the TME *via* downregulation of the expression of PD-L2 on human DCs and tumor cells and increasing tumor T cell recognition (91, 92). Paclitaxel treatment may also be beneficial for immunotherapy in ovarian cancer, as it has been shown to elevate the levels of CD8+ T cell infiltration in an ovarian cancer mouse model through increasing expression of both MHC-I and PD-L1 (93). Mkrtichyan et al. have claimed that a combination of cyclophosphamide and immune checkpoint inhibitors, such as anti–PD-1, could collaboratively decrease Treg infiltration and stimulate the yield of CD8+ TILs (94). Combining gemcitabine chemotherapy drugs with a CTLA-4 blockade could induce a potent CD4+ and CD8+ T cell–dependent antitumor immune response (95). Chemotherapy in combination with immunotherapy may be mutually beneficial, as chemotherapy can generate antigenic molecules as an *in situ* vaccine, and immunotherapy can counterbalance the acute immunosuppression induced by chemotherapy (96).

### Radiation Therapy in Combination With Immunotherapy

EOC is sensitive to radiation therapy, but abdominal radiotherapy-induced side effects, such as intestinal obstruction and ureteral stenosis or fistulae can occur, suggesting the need for close attention to dosimetry (97). Radiation therapy can enhance immunotherapy efficacy *via* inducing *in-situ* vaccination and immune reprogramming (98). Radiation therapy-induced double-strand break DNA fragments are recognized by cyclic GMP-AMP synthase, a pattern-recognition receptor that promotes the accumulation of type I interferon (99). Interferon is essential to DC recruitment and cross-activation of T effector cells, which is necessary in order to convert a tumor into an in-situ vaccine (100). Besides providing chemoattractants to attract T cells, radiotherapy also accelerates their homing into the tumor bed by increasing the expression of adhesion molecules, such as ICAM-1, on the tumor vasculature endothelium, promoting leucocyte endothelial transmigration (101). Tumor cells damaged by radiation also release damage-associated molecular pattern molecules, including high-mobility group box 1, which stimulate APCs. High-mobility group box 1 is a chromatin nuclear protein that is released mainly after necrotic cell death, and serves as a toll-like receptor 4 ligand on APCs (102). A preclinical study demonstrated that mice treated with radiotherapy in combination with CTLA-4 had increased survival compared to those treated only with radiotherapy (103). In conclusion, immunotherapy efficacy was enhanced by radiotherapy, as radiotherapy can function as an *in-situ* vaccination and accelerate T cell arrival to the tumor site.

### Anti-Angiogenesis Drugs in Combination With Immunotherapy

Bevacizumab has been shown to elevate the antitumor efficacy of cisplatin in a xenograft ovarian cancer model (104), and has been used as a first-line treatment in advanced EOC, in combination with carboplatin and paclitaxel, according to positive phase III data (105). Previous studies have shown that VEGF increases the levels of PD-1 expression in intratumoral CD8+ T cells, which could be impaired by anti-VEGF drugs (106). The combination of anti–PD-1 and VEGF-A blockade strengthened the collaborative antitumor effect in patients with high expression of VEGF, as compared with those treated with a monotherapy (107). VEGF antibodies, in combination with a tumor vaccine that facilitates granulocyte-macrophage colony stimulating factor secretion, have been demonstrated to downregulate the number of CD4+ CTLs and improve vaccine efficacy (108). However, the severe side effects of this combination therapy need to be fully evaluated. A clinical trial has reported the PD-1 inhibitor, durvalumab, in combination with endothelial growth factor receptor 1-3 inhibitor, cediranib, induced high incidence of drug-associated treatment-emergent adverse events (109). Therefore, the safety of combination therapies for ovarian cancer treatment should be given priority in further clinical trials.

### PARP Inhibitors in Combination With Immunotherapy

PARPs are a family of 17 nucleoproteins that are characterized by a common catalytic site that is involved in DNA damage repair (3, 4, 7). Germline mutations in BRCA1 and BRCA2 are harmful in ovarian cancer and other malignant tumors, making these tumors particularly sensitive to PARP inhibitors (PARPis) (110, 111). PARP inhibitors enhance tumor antigenicity, which sensitizes cancers to checkpoint blockade therapies. PARPis have also been thought to improve the response of homologous recombination-deficient ovarian cancers to immunotherapy through the generation of a higher mutation burden, which elevates the level of neoantigen expression. The stimulator of interferon genes pathway is activated by DNA damage and neoantigen expression, and plays an indispensable role in innate immunity (112). PD-L1 inhibitors have been shown to strengthen the antitumor activity of PARPis by restoring antitumor immunity (113), and showed modest clinical activity in recurrent ovarian cancer (114). Therefore, evaluating the efficacy of immune checkpoint blockade in combination with PARPis in ovarian cancer clinical trials is a potential treatment strategy. In a phase I/II TOPACIO trial, combination therapy with niraparib and pembrolizumab was evaluated for the treatment of platinum-resistant ovarian cancer (115). In the cohort with BRCA1/2 mutations, ORR and DCR were 45 and 73%, respectively. The phase I/II basket MEDIOLA trial demonstrated that olaparib in combination with an anti–PD-L1 antibody, durvalumab, for the treatment of germline BRCA 1/2 mutations that were platinum-sensitive resulted in relapse of ovarian cancer (116). At 12 weeks, DCR was 81% and ORR was 63%.

## Challenges and Future Developments

### Challenges

#### Hyperprogressive Disease

Hyperprogressive disease (HPD) is a side effect of immune checkpoint inhibitors in various types of tumors, and is associated with shorter progression-free and overall survival (117). A number of studies have reported that the incidence of HPD ranges from 4% to 29% (118–121). For ovarian cancer, retrospective analysis of data from a clinical trial with a cohort of 89 patients that received ICB showed that over half of the patients (N = 46, 51.6%) experienced early treatment discontinuation (≤12 weeks after treatment initiation) due to radiographic or clinical disease progression (122). The biological basis and mechanisms underlying HPD, such as the Fc region of antibodies (123), EGFR and MDM2/MDM4 amplification (123), and senescent CD4+ T cells (124), are being clarified. This phenomenon has polarized oncologists, who debate whether this effect could still reflect the natural history of the disease. Therefore, it is important to identify these underlying mechanisms to predict which patients are susceptible to HPD, so that it can be prevented.

#### Cancer Immunotherapy Resistance

Although the overall survival rate of ovarian cancer benefits from spontaneous anti-tumor immune responses and cytotoxic T cell infiltration, the presence of clinical disease indicates that immune effector cells are insufficient to inhibit tumor growth (125, 126). Since the interactions between the immune system and cancer cells are durable, active, and progressive, the development of an initial cancer cell into metastatic disease depends on immune evasion. There are two factors that can drive immune escape: recruitment of suppressive cells mediated by tumor cells, and iatrogenic factors such as treatment programs that include lymphotoxic drugs (126).

Immunotherapy resistance mechanisms contain primary resistance, adaptive immune resistance, and acquired resistance (127). Primary resistance is a clinical condition in which a cancer does not respond to an immunotherapeutic agent (128–130). Adaptive immune resistance occurs when a tumor has been recognized by the immune system, but is able to adapt to immune attack (131, 132). With acquired resistance, the cancer is sensitive to immunotherapy at first, but then relapses and progresses after a period of time (133–135). In the clinic, immunotherapy has been applied to solid tumors for a long time, but there are still issues that limit the development of cancer immunotherapies. First, the effect of the immunotherapies must be validated in preclinical animal models, prior to being administered to actual cancer patients in clinical trials. In most cases, immunotherapy strategies have been shown to suppress tumor development in animal models, but have often been ineffective in patients. Since the immune reaction is a complex and highly regulated biological process, the animal models used to test cancer immunotherapies do not sufficiently replicate the complex phenomenon of tumor immunity in humans. Mestas et al. have summarized the differences between mouse and human immunology in terms of both innate and adaptive immunity, including: balance of leukocyte subsets, defensins, Toll receptors, inducible NO synthase, the NK inhibitory receptor families, the B cell and T cell signaling pathway components, cytokines and cytokine receptors, T helper cells 1/T helper cells 2 differentiation, costimulatory molecule expression and function, and chemokine and chemokine receptor expression (136). Thus, discrepancies should be taken into account when using mice as preclinical models to investigate the mechanisms and efficacy of cancer immunotherapies. Since the anti-tumor efficacy of immunotherapies is long term, it is difficult to access long-term clinical efficacy in preclinical animal models. For immunotherapies, an initial antitumor effect in animal models is not synonymous with final clinical efficacy.

Collectively, a better understanding of the mechanisms of an effective antitumor response and the different intrinsic and extrinsic factors acting on the tumor cells that result in primary, adaptive, and acquired resistance to immunotherapy is needed (127).

#### Immune-Related Adverse Events

Immune-related adverse events (irAEs) are autoimmune manifestations induced by the alteration of the immune system *via* immunotherapy treatment, such as checkpoint inhibitors and adoptive cell therapy (137). IrAEs have a high incidence in multiple types of cancers, with anti-CTLA-4 therapy, ipilimumab, and anti–PD-1 or anti–PD-L1 therapies, at 90 and 70%, respectively. Studies suggest that immune-related adverse events (irAEs) may develop through an integrated pathway, including autoreactive T cells, autoantibodies, and cytokines (138). For instance, T cell infiltration in tumor tissue induces T cell activation and leads to the production of inflammatory cytokines, which promote the development of irAEs (138). Immune-related adverse events often affect a range of organs, including skin, colon, endocrine glands, lungs, and liver. Mild effects that can be managed *via* transitory immunosuppression therapy with corticosteroids account for the majority of adverse events; however, severe events often lead to hospitalization and require specialized treatment. Patients, nurses, and other collaborative staff must be educated on these adverse events when considering the use of these drugs for the treatment of cancer (138).

### Future Development

#### Single-Cell Technologies Could Explain the Functional Heterogeneity of Immune Processes

Single-cell genomics is an advanced technology that could revolutionize the way we evaluate complex immune cell assemblies and explore their spatial organization, dynamics, clonal distribution, pathways, function, and crosstalk (139). Krieg et al. utilized high-dimensional single-cell mass cytometry and a bioinformatics pipeline to qualify the immune cell subsets in the peripheral blood of patients with melanoma, before and after anti–PD-1 immunotherapy. They found that CD14+CD16−HLA-DRhi monocytes are a strong predictor of progression-free and overall survival in response to anti–PD-1 immunotherapy (140). Multi-omic datasets at single-cell resolution, in combination with advanced computational methods, will improve the determination of immune cell identity. Current datasets, integrated with ‘big data’ methodologies, can serve as a platform to sustain future immunology research. Thus, in the future, these methods may apply to functional studies of immune cell populations and precision medicine (141). Azizi and his colleagues have demonstrated that combining single-cell analysis of the tumor immune microenvironment in breast cancer with computational analysis can result in the production of an immune map of breast cancer (BC) that points to continuous T cell activation and differentiation states (142). Single cell analysis was also used to reveal the association between CAF clusters (CAF-S1) and immunotherapy resistance. These findings indicated that there is a positive feedback loop between specific CAF-S1 clusters and Tregs that plays a vital role in immunotherapy resistance (143). Recently, single-cell RNA sequencing (scRNAseq) computational analyses were applied to melanoma tumors to investigate the tumor cell states that promote immune evasion. Using this novel technology, a resistance program that is expressed by tumor cells has been identified, which is involved in T cell exclusion and immune evasion (144). With the development of single-cell technology, it is possible that immunotherapy will have fewer adverse events and a more effective response, based on modified patient stratifications, identification of novel biomarkers, and identification of novel cell targets and pathways (145).

#### Nanotechnology Could Strengthen the Efficacy of Immunotherapy

Nanotechnology is critically important for immunotherapy for several reasons, including its potential to enhance efficacy, it can be translated, and it can improve novel therapeutic strategies that are based on current cancer immunotherapies (146). In the near future, we envision that nanotechnology will be a key driver of cancer immunotherapy success. Jin et al. have identified that more adaptive and durable responses and more robust antitumor effects will enhance the effects of immunotherapeutic cells through nano-immunoengineering *via* regulation of the immune-network and identification of precise cancer-targeted theranostics (147). Nanomedicines can also regulate myeloid and lymphoid cell behavior, consequently strengthening anticancer immunity and immunotherapy efficacy (148, 149). Nanoparticles that are used as advanced biomaterials could enhance the efficacy of immunotherapies and reduce harmful side effects (150). Nanoparticles can be applied to reprograming the immunosuppressive tumor microenvironment and triggering systemic antitumor immunity, coupled with immunotherapy agents against advanced cancer (151, 152).

In ovarian cancer, nanotechnology-based immune checkpoint inhibitor delivery systems have the potential to overcome the immunosuppressive environment and transport barriers. This have already been utilized to improve the distribution and targeting-capabilities of drugs against tumor-associated immune cells, such as DCs and macrophages (153). Radiotherapy is frequently applied for the treatment of various kinds of cancer. However, the limitations of radiotherapy are resistance induced by tumor tissue hypoxia and uncontrollable metastases. To address these issues, scientists have designed core–shell nanoparticles that are composed of an enzyme that can decompose H_2_O_2_ to generate O_2_ and a toll-like-receptor-7 agonist that can regulate the immune suppressive tumor microenvironment.(154).

#### The 3D-Organoid Model Can Model the Tumor-Immune Microenvironment

Organoids that simulate the structure and function of their *in vivo* counterpart organs are grown from stem cells *in vitro*. This technique has been used as a novel human cancer treatment, as described in our previous studies (155–158). Tumor organoids, in combination with immune cells and fibroblasts, can be utilized for immune-oncology applications (159). Recently, an air-liquid interface (ALI) method has been used to fabricate patient-derived organoids (PDOs) with native embedded immune cells (T cells, B cells, NK cells, and macrophages) that enables investigation of the tumor microenvironment and personalized immunotherapy testing (160).

Hill et al. have used organoid models that are defective in homologous recombination (HR) and replication fork protection for drug sensitivity screening. They found that HR deficiency is related to PARP inhibitor sensitivity, and that replication fork protection deficiency is correlated with carboplatin and CHK1 and ATR inhibitor sensitivity (161). Schnalzger et al. have developed a three-dimensional (3D) patient-derived colon organoid to evaluate CAR efficacy and tumor specificity in a personalized manner. This new preclinical model allowed testing of CAR-mediated cytotoxicity in a tissue-like environment, but further basic and clinical trials are needed to confirm these findings (162). Recently, a novel organoid composed of cells derived from lymph node and tumor tissue from the same patient was developed to evaluate the efficacy of immunotherapy and to assess the relationship between the clinical response of the patient to therapy (163). Lymph node stromal cells (LNSCs) have been reported to be involved in the inhibition of early activation of autoreactive immune cells and peripheral tolerance (164). Mechanisms can be clarified using this advanced organoid model.

#### Emerging Biomarkers for Immuno-Oncology

Cancer immunotherapies can be grouped into two categories based on the presence or absence of a suppressed immune response to each patient’s tumor. If immunotherapy can trigger a prior immune response, patients are eligible to choose checkpoint inhibitor drugs to trigger the prior immune response and kill tumor cells. Whereas, if patients do not have a prior immune response, then a checkpoint inhibitor will have no effect on the activity of the immune response. Strategies that stimulate a new immune response are more appropriate in these cases.

Immunohistochemistry (IHC) testing to detect the expression of PD-L1 has been one of the first predicted biomarkers for pembrolizumab efficacy (165). However, about 15% of patients that showed PD-L1 negativity exhibit a response to the treatment with PD-1 or PD-L1. The IHC test was also used to evaluate the treatment efficacy of cancer vaccines or with bispecific T cell redirection therapies. Detection of expression of the antigen in the patient for the cancer vaccine or the tumor-specific antigen on the bispecific molecule is necessary.

Interleukin-8 has been reported as a poor predictor of outcome of immune checkpoint blockade in urothelial carcinoma (mUC) and metastatic renal cell carcinoma (166). Anti–PD-1 monoclonal antibody therapy (atezolizumab) showed poor therapeutic effect with high levels of IL-8 in plasma, peripheral blood mononuclear cells, and tumors. A large-scale retrospective analysis reported that the poor prognoses of advanced cancer patients treated with nivolumab and/or ipilimumab, everolimus, or docetaxel in phase III clinical trials were related to upregulated baseline serum IL-8 (167). The effect of IL-8 in ovarian cancer remains unclear, and further investigation is warranted. Molecular analysis of the T cell repertoire (168) could digitize the immune response. Patients with high T cell infiltration in solid tumors and high clonality had a significantly increased response to PD-1; however, patients with low T cell infiltration and no evidence of clonal T cell expansion had a poor response.

Next-generation high-throughput DNA (NGS) sequencing techniques have provided new opportunities for immunotherapy. The high-throughput and deep coverage of NGS techniques could help with whole exome sequencing or RNA sequencing to identify somatic mutations that may encode neoantigens (169). These neoantigens may be used to customize cancer vaccines and predict the response to immunotherapy. Recent studies have reported that recognition of neoantigens is a crucial factor in the activity of clinical immunotherapies, and cutting-edge technologies enable the dissection of the immune response to patient-specific neoantigens. These findings indicate that neoantigens may form a biomarker in cancer immunotherapy and provide novel insights into the development of enhanced T cell efficacy against this class of antigens (85).

## Discussion

Epithelial ovarian cancer (EOC) has early metastasis, peritoneal dissemination, and omentum infiltration. The omentum is an organ rich in lipids, which play a vital role in EOC. Lipid metabolic disorder of ovarian cancer cells, characterized by the alteration of lipid uptake and lipogenesis, are involved in EOC metastasis, alterations of ovarian cancer stem cells, chemotherapy resistance, and immunotherapy (170). Interestingly, for the immune system, alteration of lipid metabolism also has impact on T cells, TAMs, regulatory T cells, and MDSCs. Veglia et al. have reported that the fatty acid transport proteins that are upregulated in MDSCs, result in immunosuppression in tumors. Deletion of fatty acid transport proteins ablated the suppressive activity of MDSCs. Combining an inhibitor of fatty acid transport proteins with checkpoint inhibitors suppressed tumor progression in mice (171). Thus, with omentum infiltration in ovarian cancer, immunotherapy strategies, in combination with agents that target lipid metabolism, is a new direction that can be explored in cancer treatment.

Ovarian cancer is a solid cancer which is still a clinical challenge for CAR-T therapy. Challenges in CAR-T cell therapy are mainly due to antigen heterogeneity, physical barriers, and the complex network of the tumor microenvironment (78). To address the issue of heterogeneity of antigen expression, next-generation CAR-T cells that target more than one antigen, such as EGFR, HER2, and IL13Rα2, have been examined in pre-clinical models (172, 173). Combining CAR-T cell therapy with epigenetic drugs that can promote the expression of target antigens is also an option to deal with antigen heterogeneity (174). In order to overcome the physical barrier, there are three strategies as reported by Fucá (78): local delivery, overcoming the aberrant tumor vasculature, and enhancing trafficking. The TME is a complex network composed of extracellular matrix and stromal cells that are associated with reduced efficacy of CAR-T therapy. Overexpression of the FasL ligand (CD95L) in the TME interacts with the Fas (CD95) receptor that is expressed in CAR-T cells and can lead to T cell apoptotic death (72). Immunosuppressive catabolites, such as adenosine soluble catabolites (175), inhibitory factors rich in TME (176), and cytokines, such as IL15 (177), have direct or indirect effects on survival, expansion, and the anti-tumor function of CAR-T cells. In order to solve the problem of solid tumors and immune surveillance, translational, cooperative, and interdisciplinary efforts are required.

**Figure 3:**
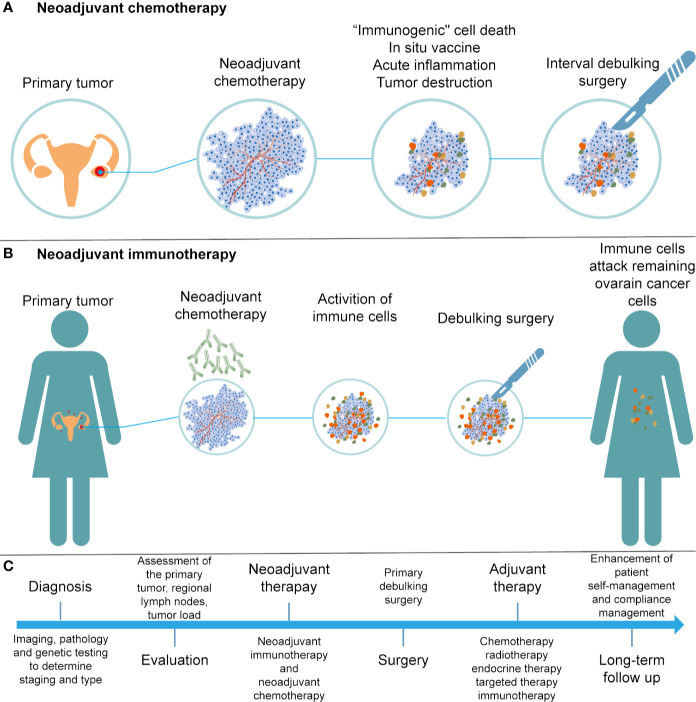
Development of treatments for ovarian cancer. **(A)** Illustration of NACT. **(B)** Illustration of neoadjuvant immunotherapy. **(C)** Flow chart of diagnosis and treatment for ovarian cancer.

Neoadjuvant chemotherapy (NACT) has been applied in patients with stage IIIC or IV ovarian cancer, who are not suitable for primary debulking surgery (PDS). Research suggests that NACT, followed by interval debulking surgery (IDS) and adjuvant chemotherapy, was not inferior to primary debulking surgery followed by chemotherapy (178). NACT may exert multiple influences on the immune system, including induction of “immunogenic” cell death, presentation of neoantigens, and an increase in acute inflammatory and tumor-destructive responses (179–181) (**Figure 3A**). In 2017, Balkwill et al. evaluated the effect of NACT on immune activation in ovarian cancer, and found that NACT may enhance the host immune response; however, the response was weakened by upregulation of PD-1 (182). NACT can amplify the TIL responses but fails to turn TIL-negative cases into TIL-positive cases in ovarian cancer (183). Thus, a personalized treatment protocol of NACT and immunotherapy should be evaluated according to the baseline features of the tumor microenvironment.

Neoadjuvant immunotherapy has demonstrated remarkable efficacy in cancer patients as compared to surgery followed by adjuvant immunotherapy, as the adverse effects of surgery-induced metastasis are associated with innate and adaptive immunity damage (184) (**Figure 3B**). Early preclinical trials in melanoma (185), non–small cell lung carcinoma (186), and glioblastoma (187) have shown that neoadjuvant immunotherapy was better than adjuvant immunotherapy in promoting clinical efficacy. Neoadjuvant immunotherapy could enhance systemic immunity against tumor antigens and eliminate micrometastases that can be considered as the source of recurrence (188). With a comprehensive understanding of efficacy and safety, neoadjuvant therapies are expected to bring substantial benefits to patients suffering from cancer.

## Conclusion

Immunotherapy is a revolution in ovarian cancer management. We present a flow chart that describes the treatment of patients with EOCs (**Figure 3C**). Despite the promising treatments that have been developed for cancer immunotherapy, such as immune checkpoint inhibitors and CAR-T therapies, there is still a need to overcome the immunosuppressive tumor microenvironment in order to improve the efficacy of cancer immunotherapy. The tumor immune microenvironment is an important regulator of immune suppression and immune tolerance, and can destroy the number and activity of TILs. A better understanding of the relationship between the tumor and stromal environment in EOCs is crucial to identify effective treatment methods and reliable predictive biomarkers. In conclusion, with an increased understanding and advanced technology, such as 3D-organoid models and single-cell technologies, more sophisticated and personal immunotherapy treatments based on tumor biology and TME characteristics can be applied in clinical practice. Thus, these efforts will enhance the benefits of immunotherapy to more patients with EOC and allow them to benefit from the long-lasting responses of immunotherapy.

## Author Contributions

W-LJ and GL designed the manuscript. CY, Y-JZ, and B-RX wrote the manuscript. CY and Z-CZ drew the figures and tables. W-LJ mainly revised the manuscript. GL made some revisions of the review. All authors contributed to the article and approved the submitted version.

## Funding

This work was supported by the National Natural Science Foundation of China (nos. 81872507 and 81872430), the Harbin Medical University (CN) Research and Innovation Project (YJSKYCX2019-55HYD), and the Special Fund in China Postdoctoral Science Foundation (2019T120281).

## Conflict of Interest

The authors declare that the research was conducted in the absence of any commercial or financial relationships that could be construed as a potential conflict of interest.

## Abbreviations

ACT, Adoptive cell therapy; CAF, Cancer-associated fibroblast; CAR, Chimeric antigen receptor; CIK, Cytokine-induced killer; CTL, Cytotoxic T lymphocyte; CTLA4, Cytotoxic T lymphocyte antigen 4; DC, Dendritic cell; EMT, Epithelial-mesenchymal transition; EOC, Epithelial ovarian cancer; HPD, Hyperprogressive disease; ICI, Immune checkpoint inhibitors; irAEs, Immune-related adverse events; MDSC, Myeloid derived suppressor cells; MHC, Major histocompatibility complex; MISIIR, Müllerian inhibiting substance type 2 receptor; NK, Natural killer; OC, Ovarian cancer; PARP, Poly-ADP-ribose polymerase; TAA, Tumor-associated antigens; TAM, Tumor-associated macrophages; TCR, T cell receptors; TME, Tumor microenvironment; Treg, Regulatory T cell.
